# Supplementary material for: One-Pot Multicomponent Synthesis and Bioevaluation of Tetrahydroquinoline Derivatives as Potential Antioxidants, α-Amylase Enzyme Inhibitors, Anti-Cancerous and Anti-Inflammatory Agents
Source: Molecules. 2020 Jun 11;25(11):2710. doi: 10.3390/molecules25112710 (PMC7321408; doi:10.3390/molecules25112710)
Supplement: Supplementary file 1 [file molecules-25-02710-s001.pdf]

One-Pot Multicomponent Synthesis and Bioevaluation of Tetrahydroquinoline Derivatives as Potential Antioxidants, α-Amylase Enzyme Inhibitors, Anti-Cancerous and Anti-Inflammatory Agents.

Samra Farooq[1,] , Aqsa Mazhar [1], Areej Ghouri[1], Ihsan-Ul-Haq[1] and Naseem Ullah [1,*]
[1] Department of Pharmacy, Faculty of Biological Science, Quaid-I-Azam University Islamabad, 45320, Pakistan
samrafarooq@bs.qau.edu.pk (S.F.,); aqsamazhar1947@gmail.com (A.M.,); areejkhan27@gmail.com (A.G.,); ihaq@qau.edu.pk (I.H.,)
* Correspondence:
Naseem Ullah (N.U.,)
Assistant Professor
nullah@qau.edu.pk ; Tel: +92-334-5023566

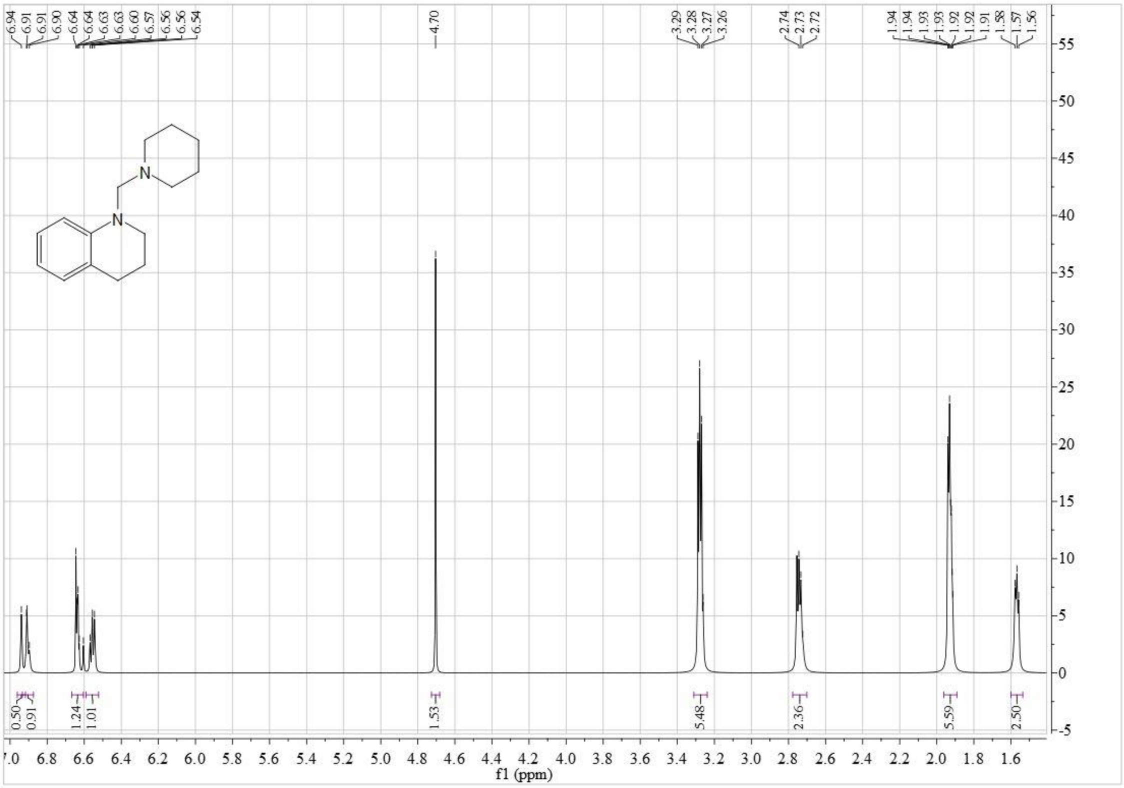

Figure S1: Proton NMR of compound SF1

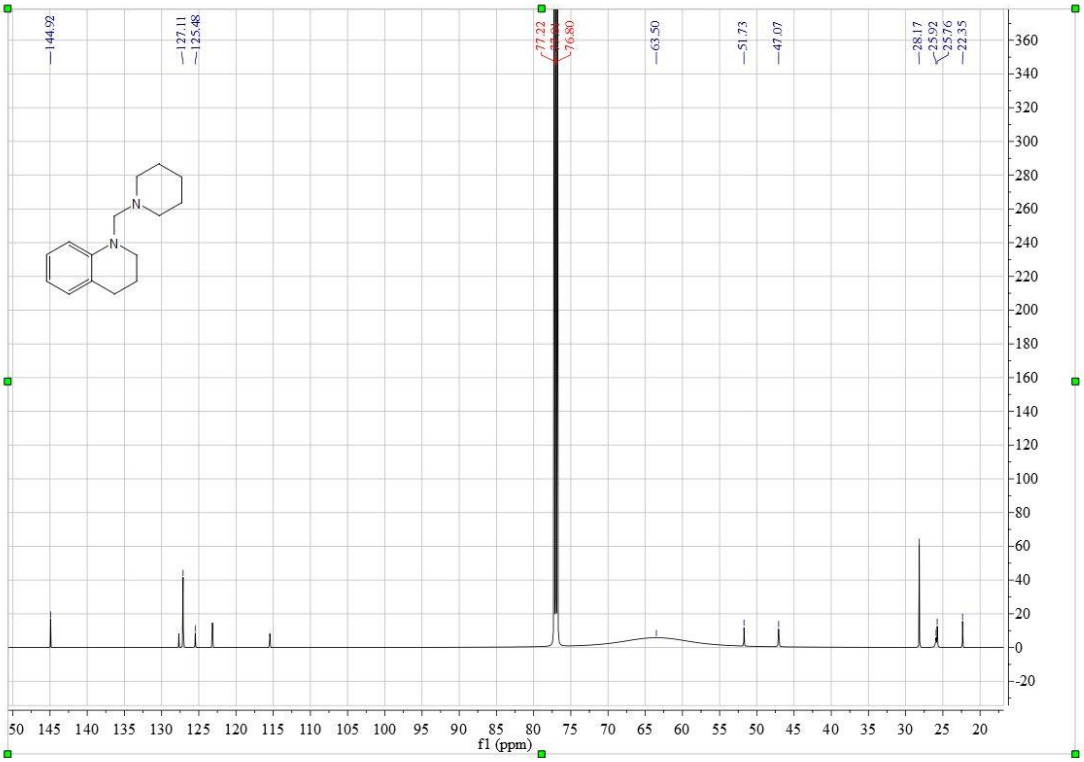

Figure S2: Carbon NMR of compound SF1

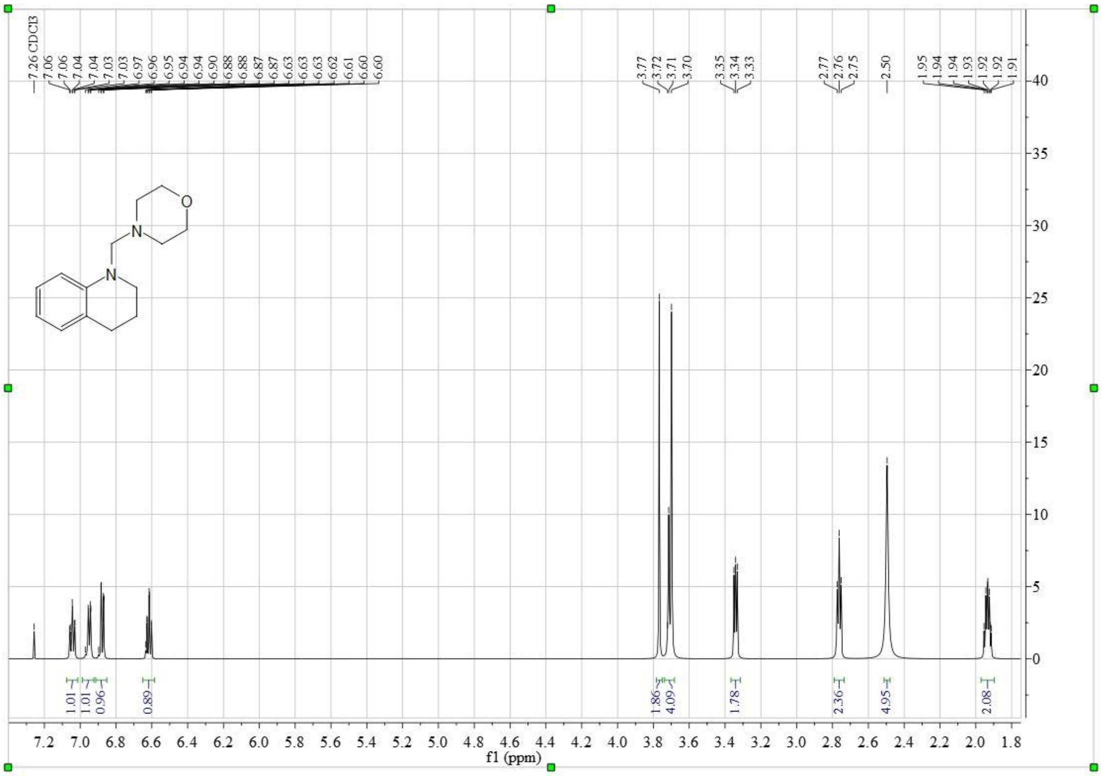

Figure S3: Proton NMR of compound SF2

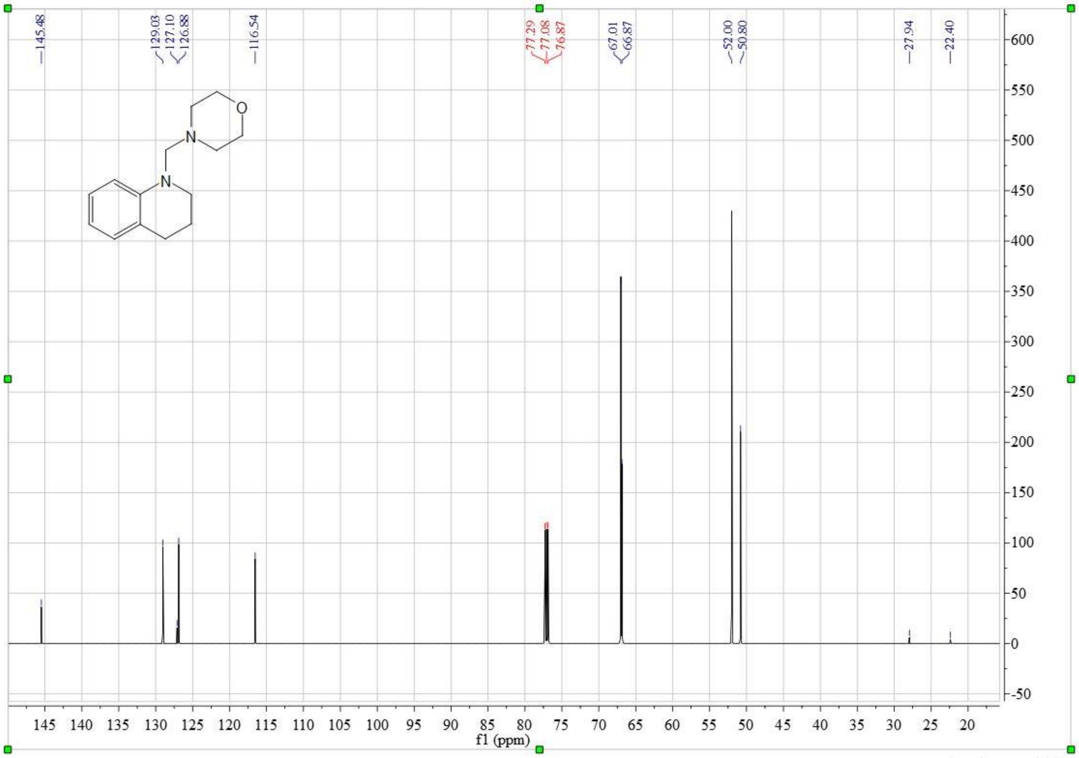

Figure S4: Carbon NMR of compound SF2

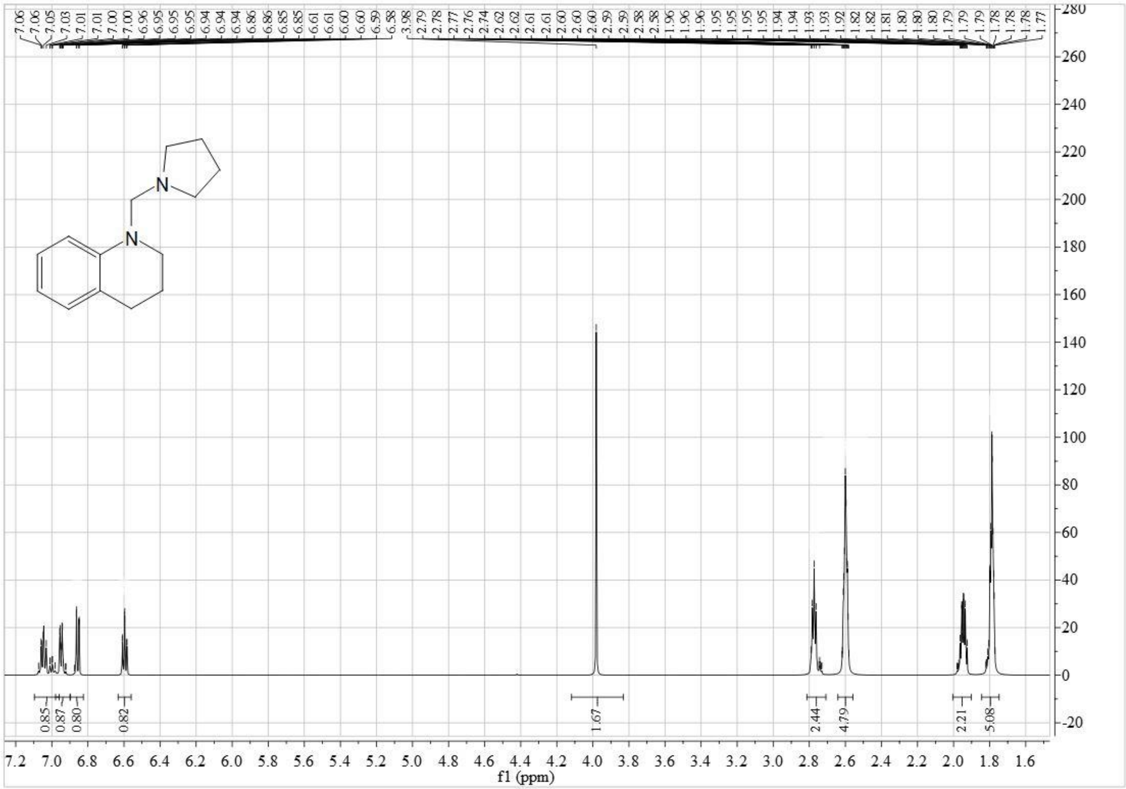

Figure S5: Proton NMR of compound SF3

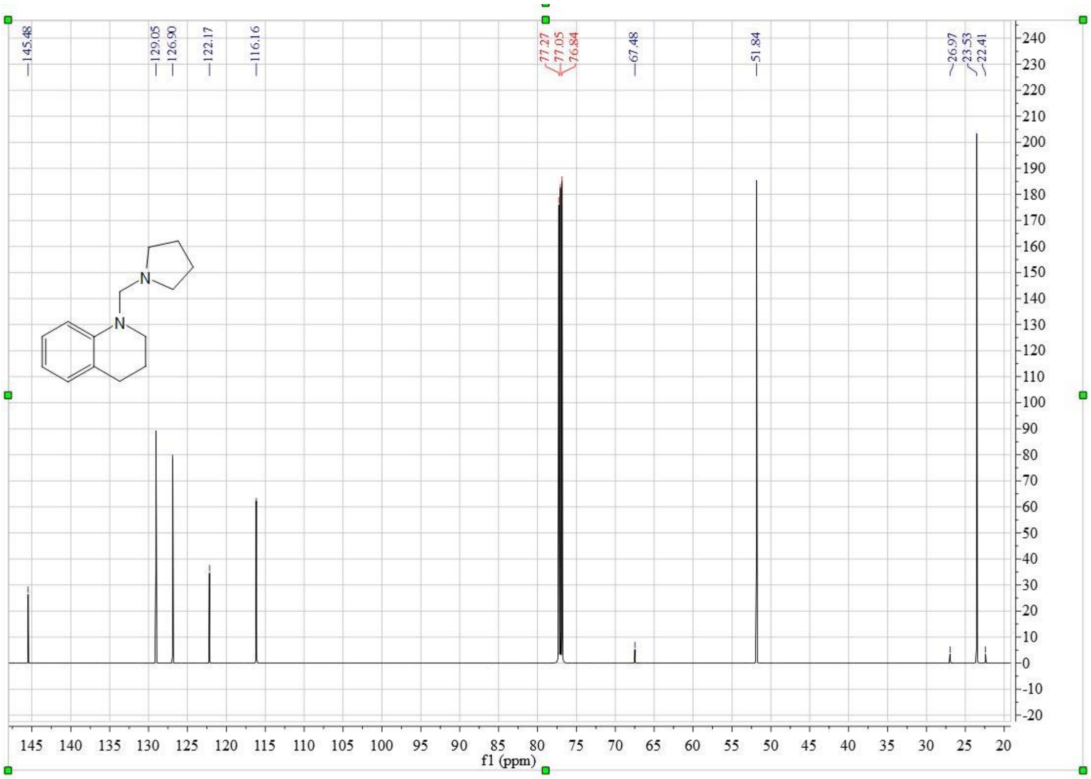

Figure S6: Carbon NMR of compound SF3

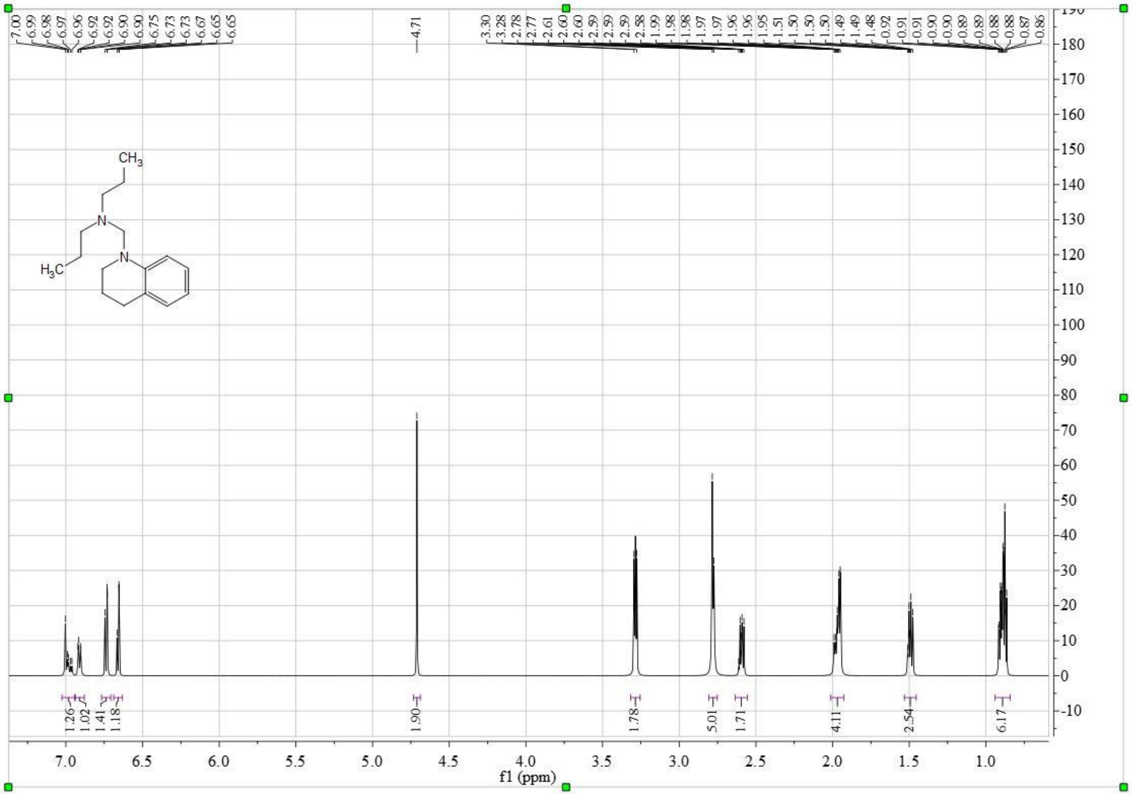

Figure S7: Proton NMR of compound SF4

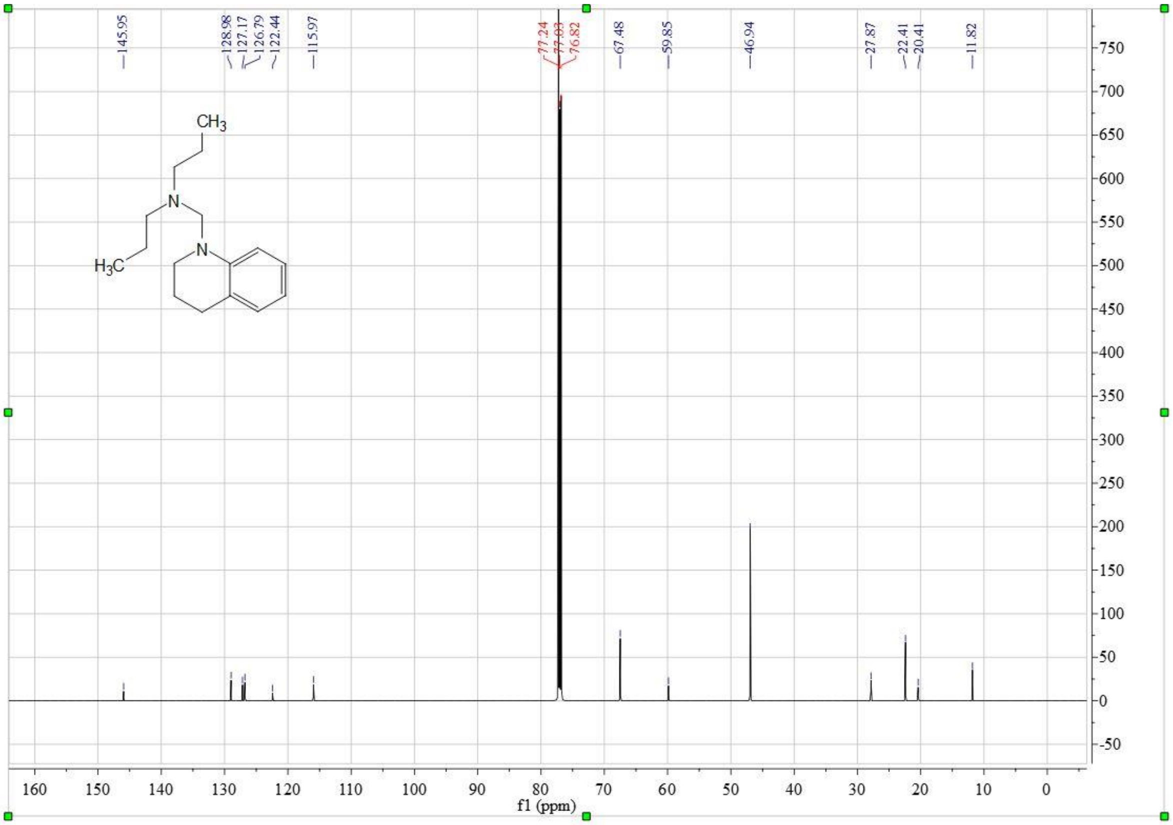

Figure S8: Carbon NMR of compound SF4

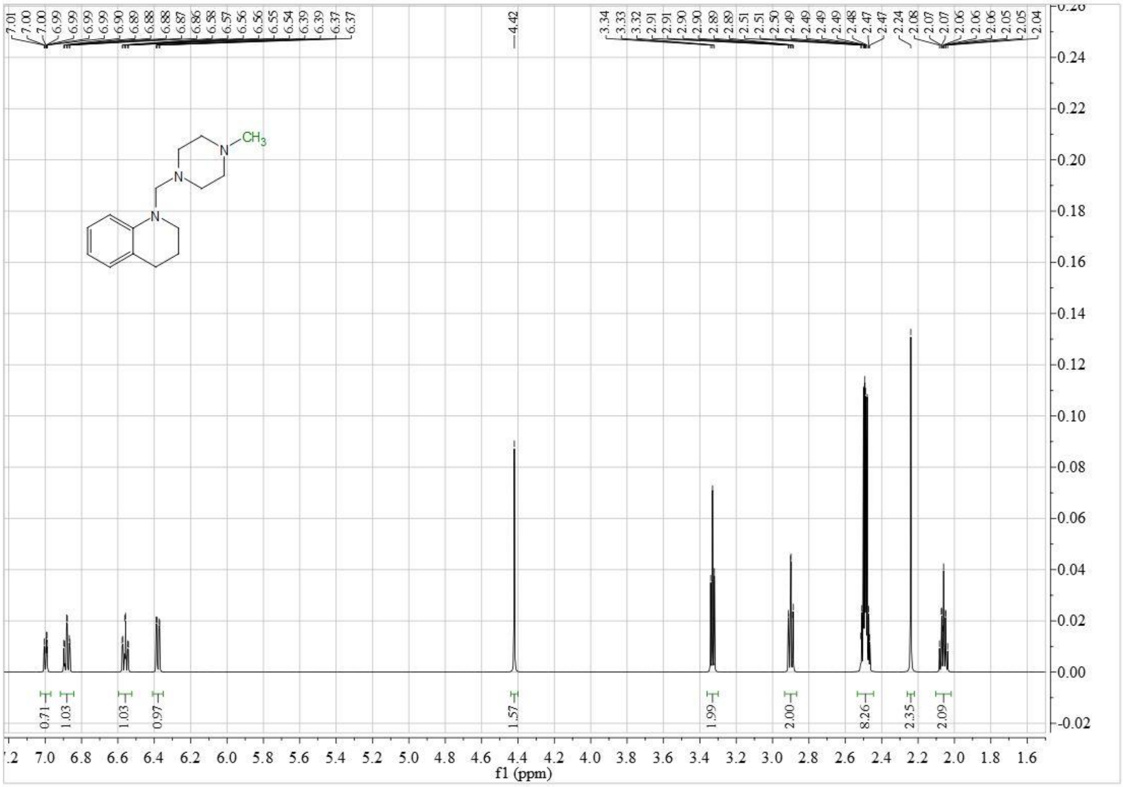

Figure S9: Proton NMR of compound SF5

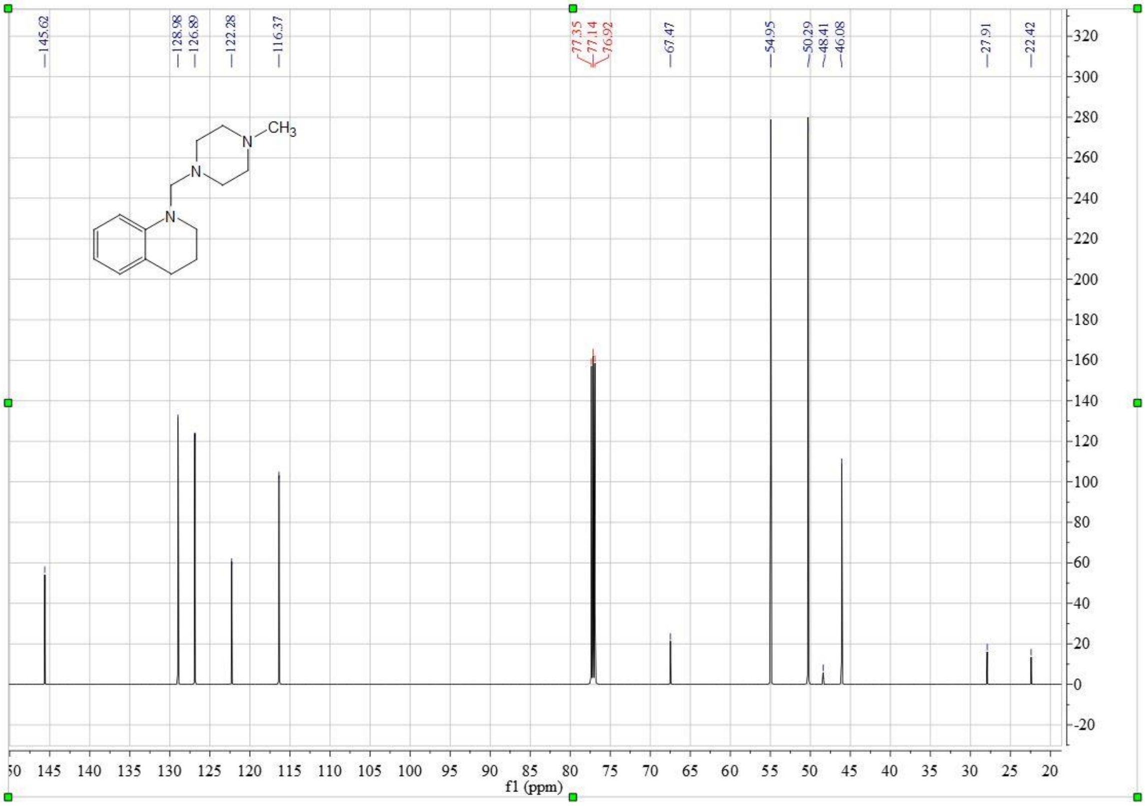

Figure S10: Carbon NMR of compound SF5

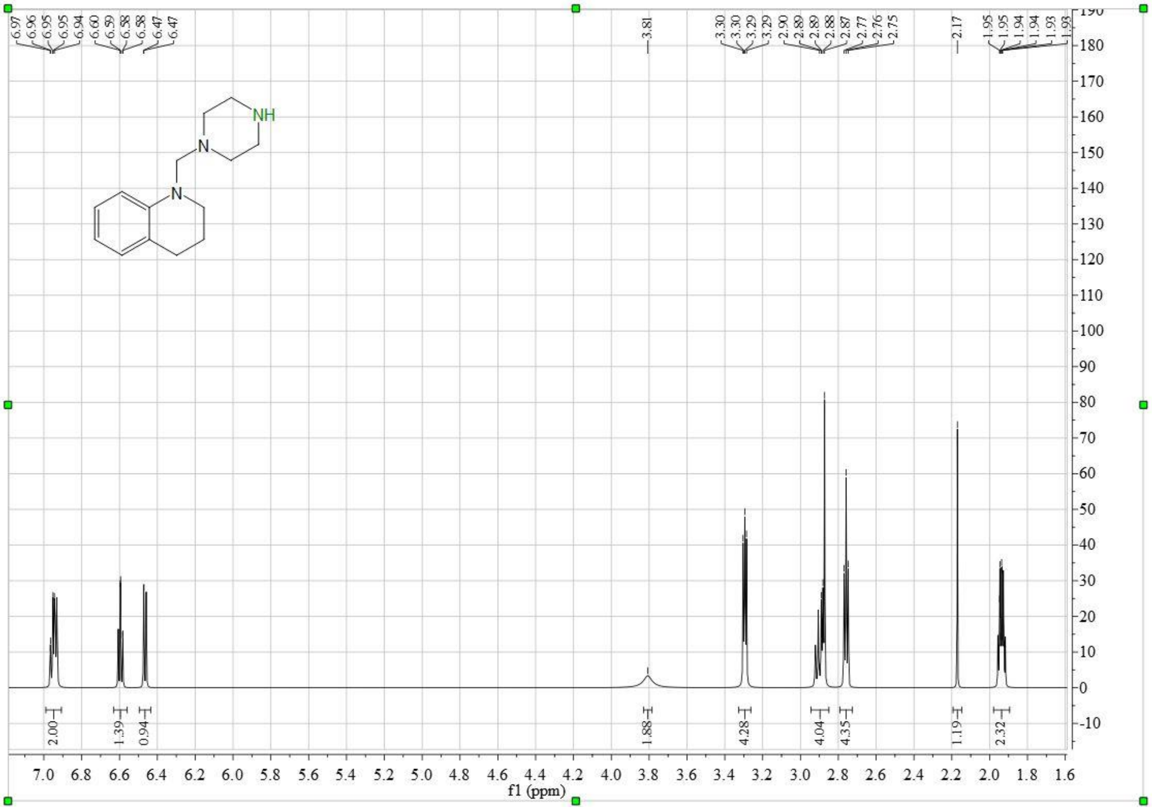

Figure S11: Proton NMR of compound SF6

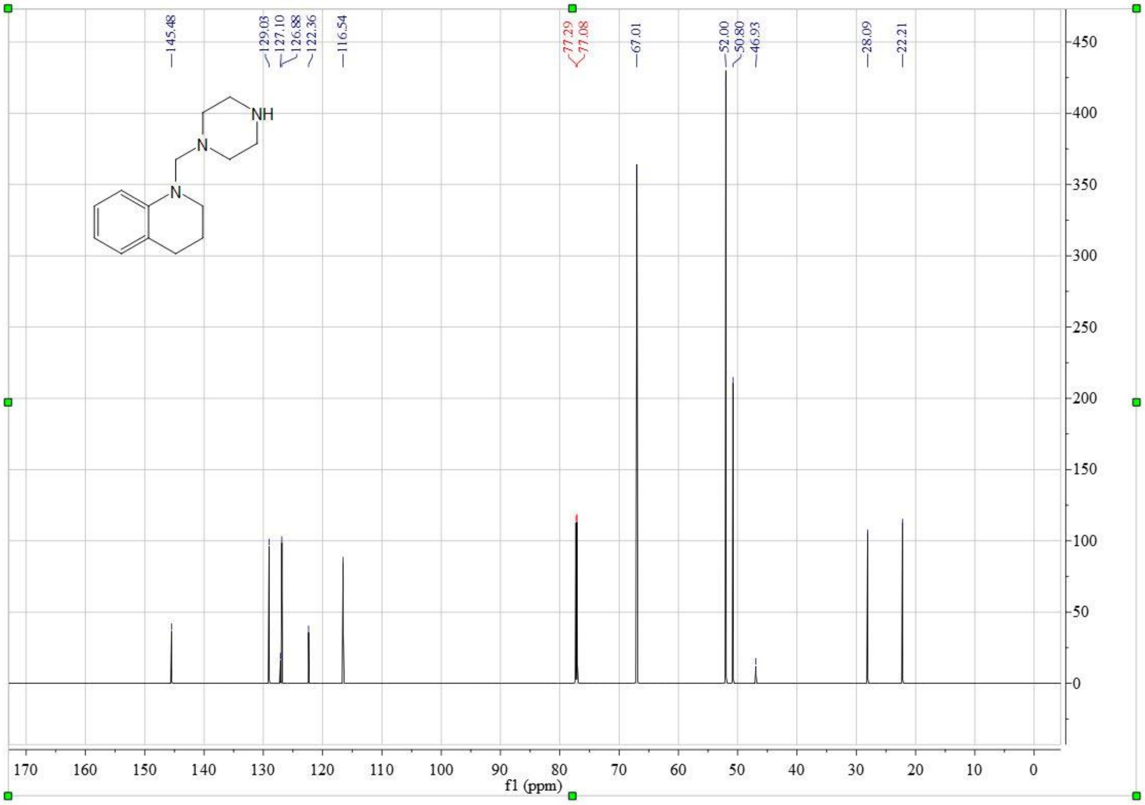

Figure S12: Carbon NMR of compound SF6

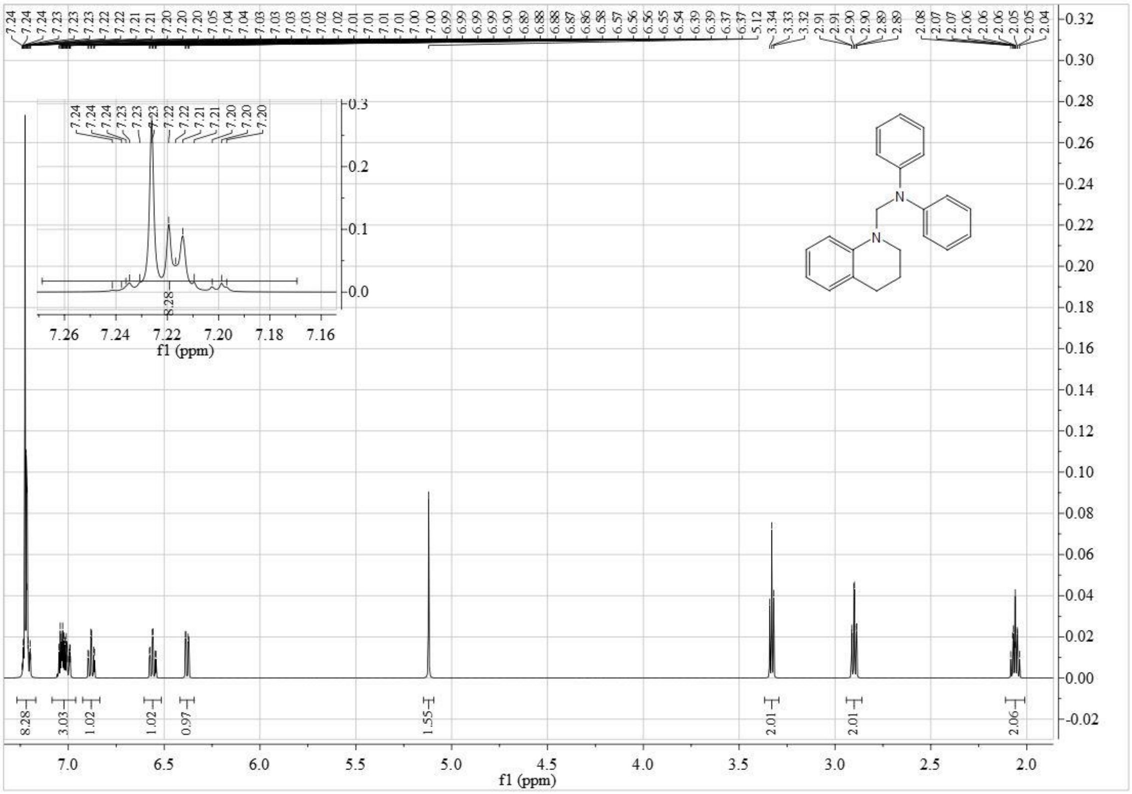

Figure S13: Proton NMR of compound SF7

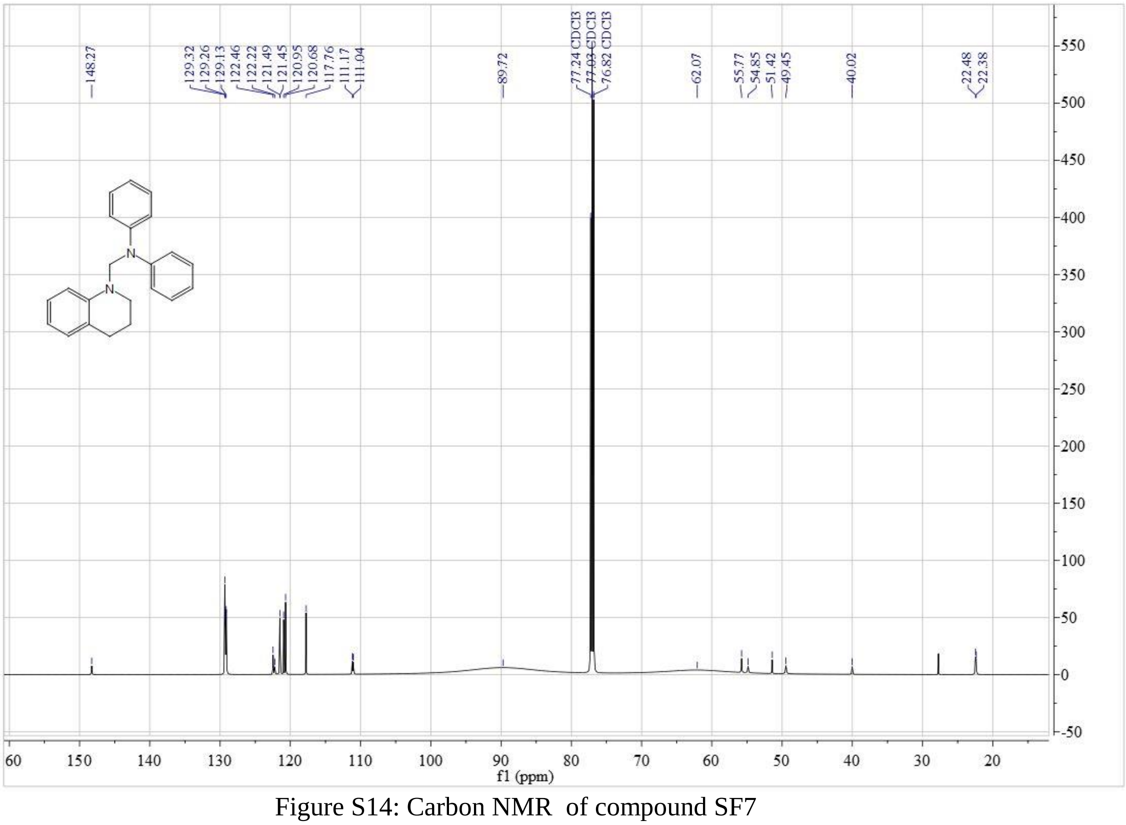

Figure S14: Carbon NMR of compound SF7

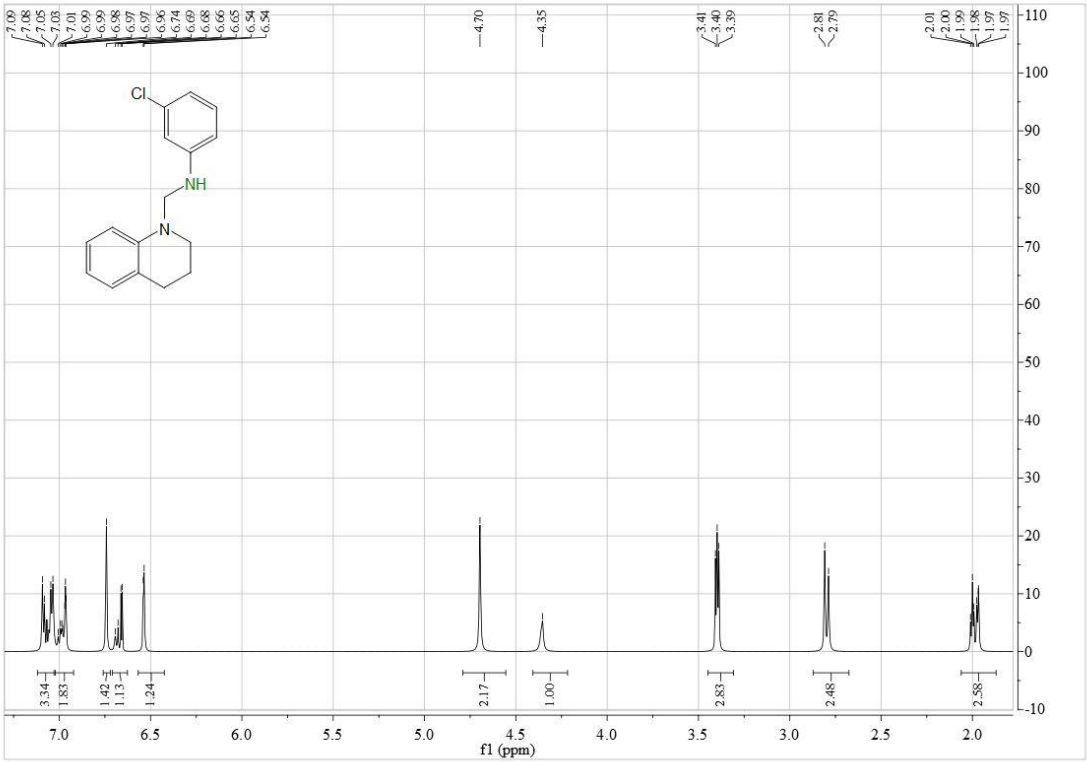

Figure S15: Proton NMR of compound SF8

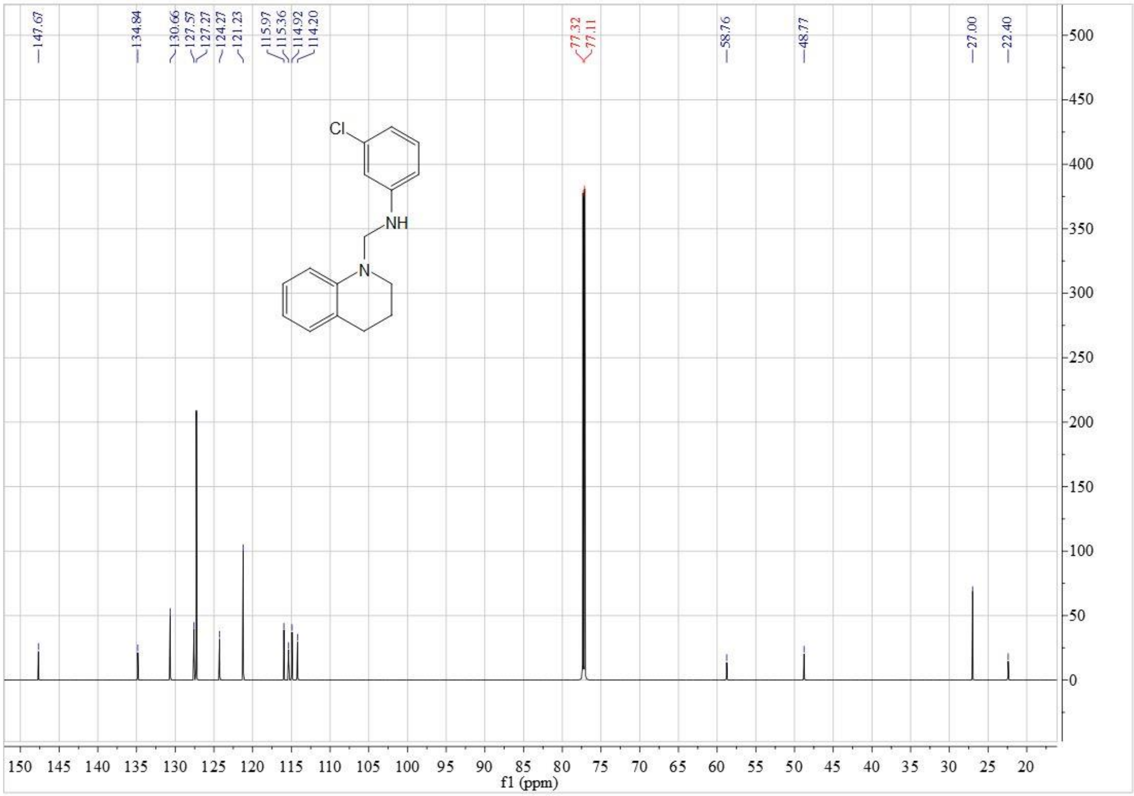

Figure S16: Carbon NMR of compound SF8

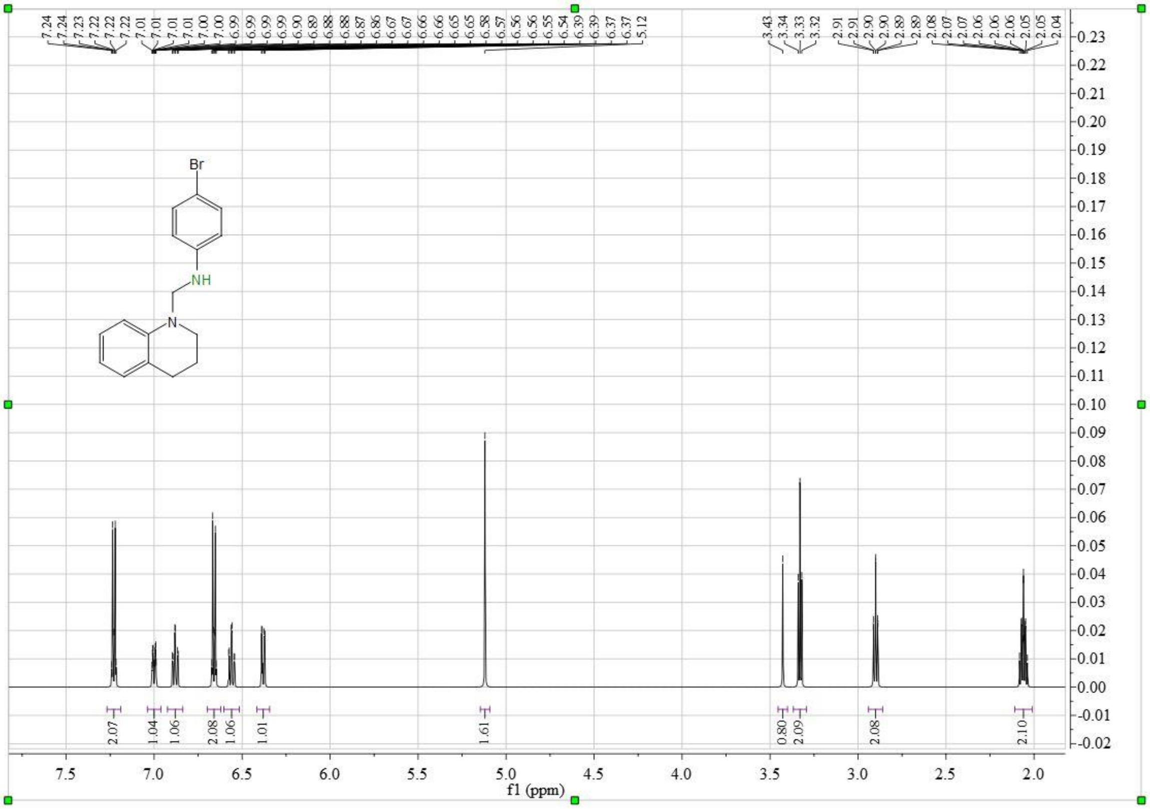

Figure S17: Proton NMR of compound SF9

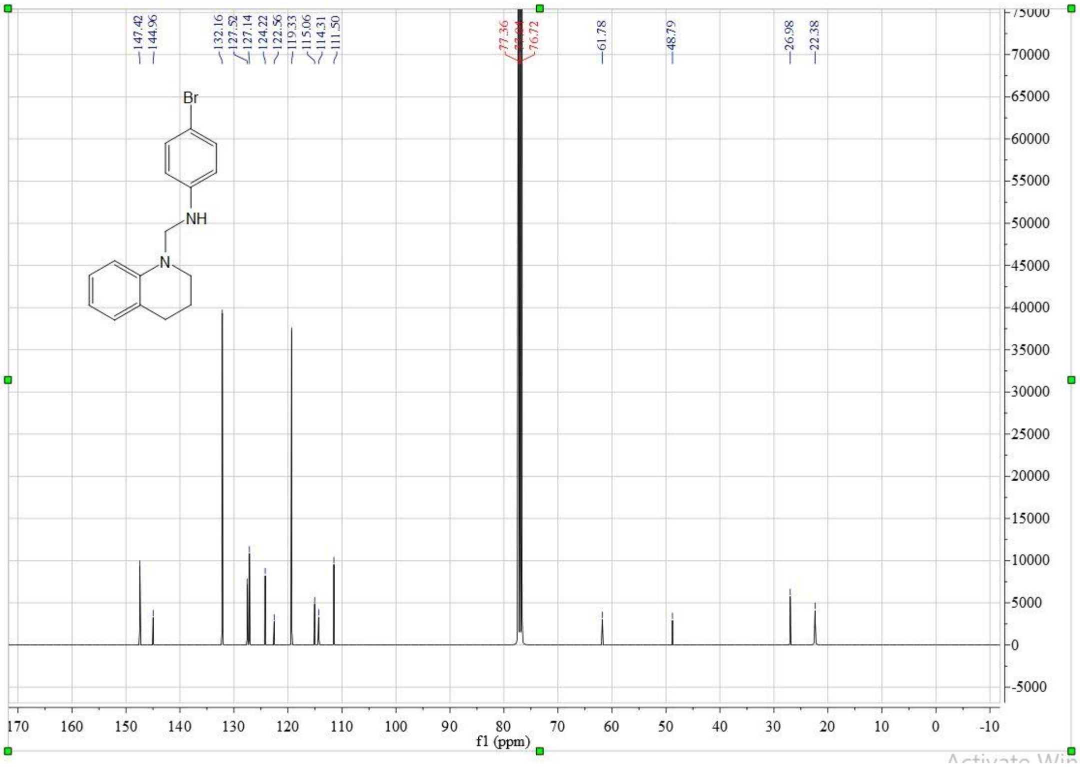

Figure S18: Carbon NMR of compound SF9

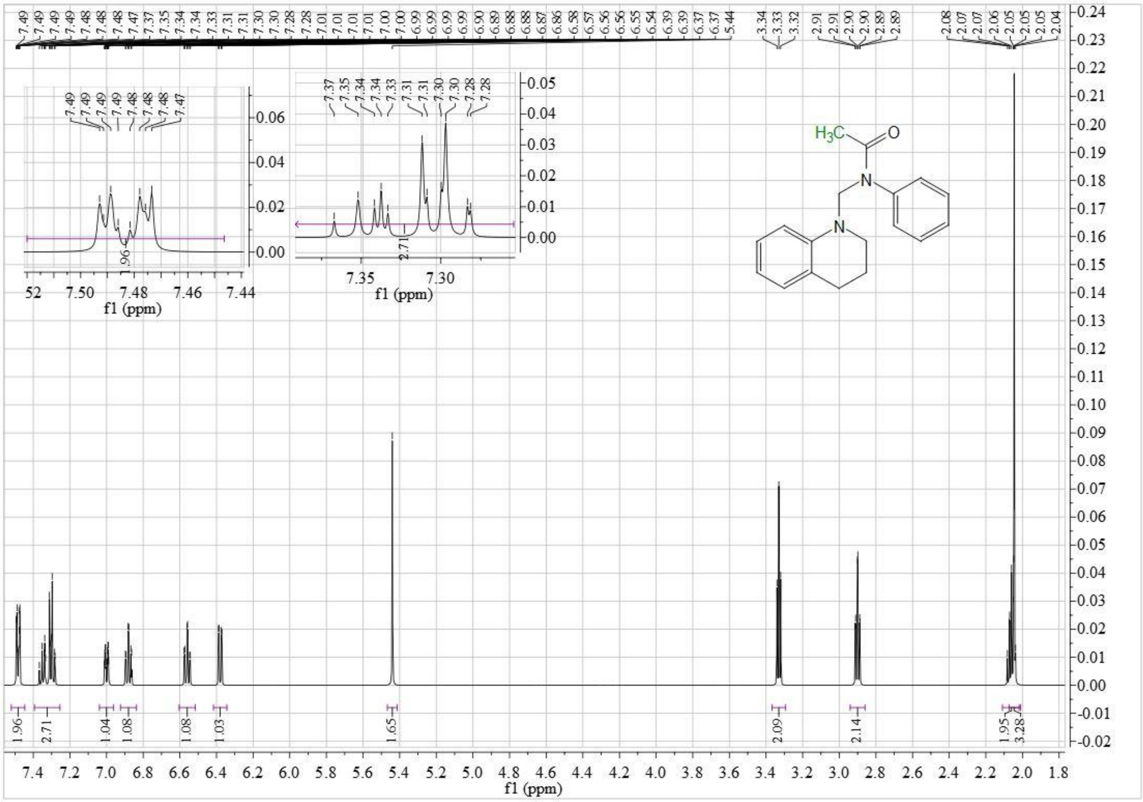

Figure S19: Proton NMR of compound SF10

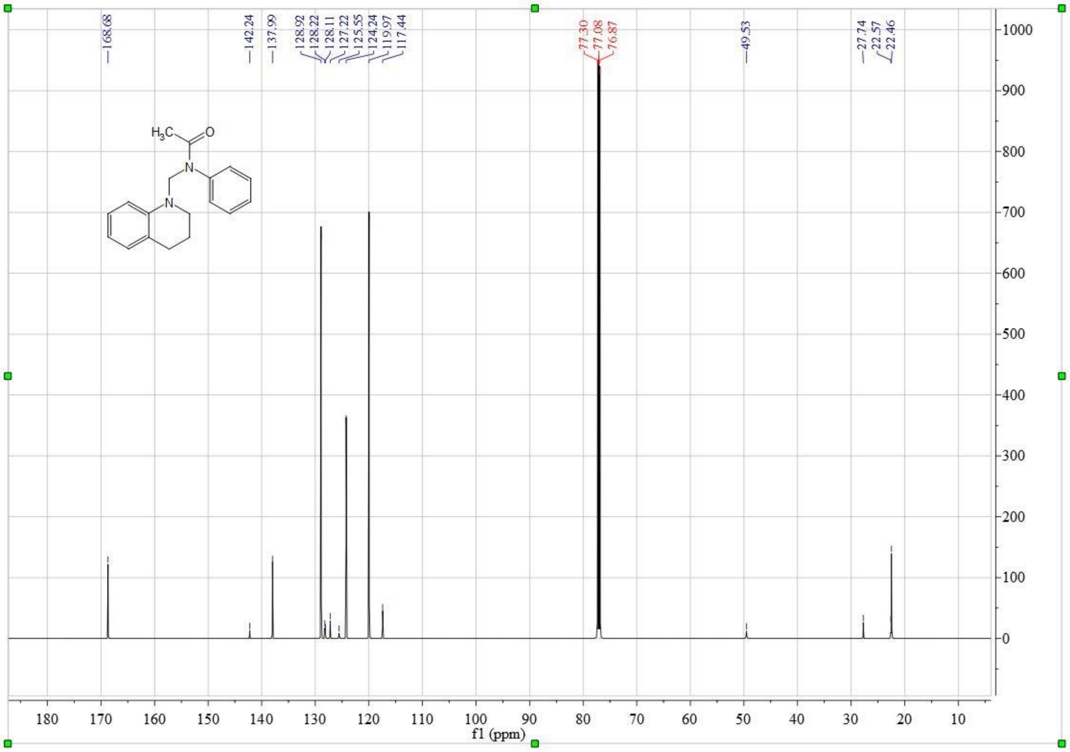

Figure S20: Carbon NMR of compound SF10

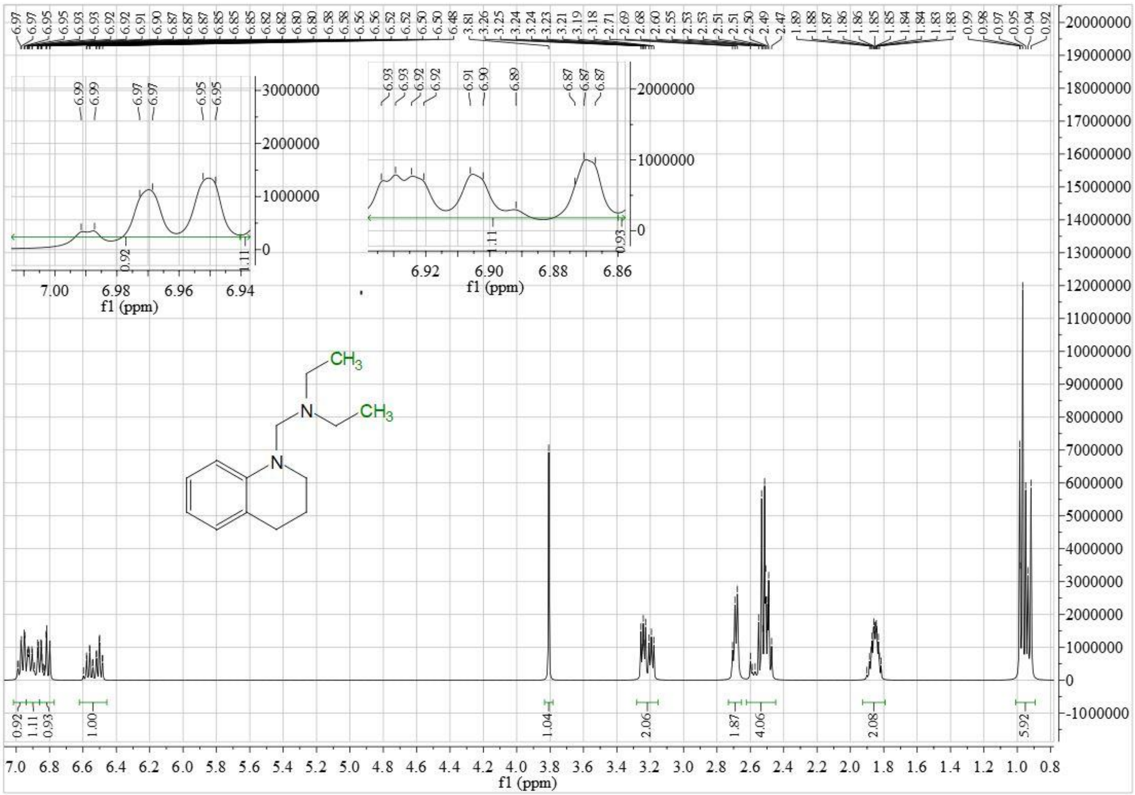

Figure S21: Proton NMR of compound SF11

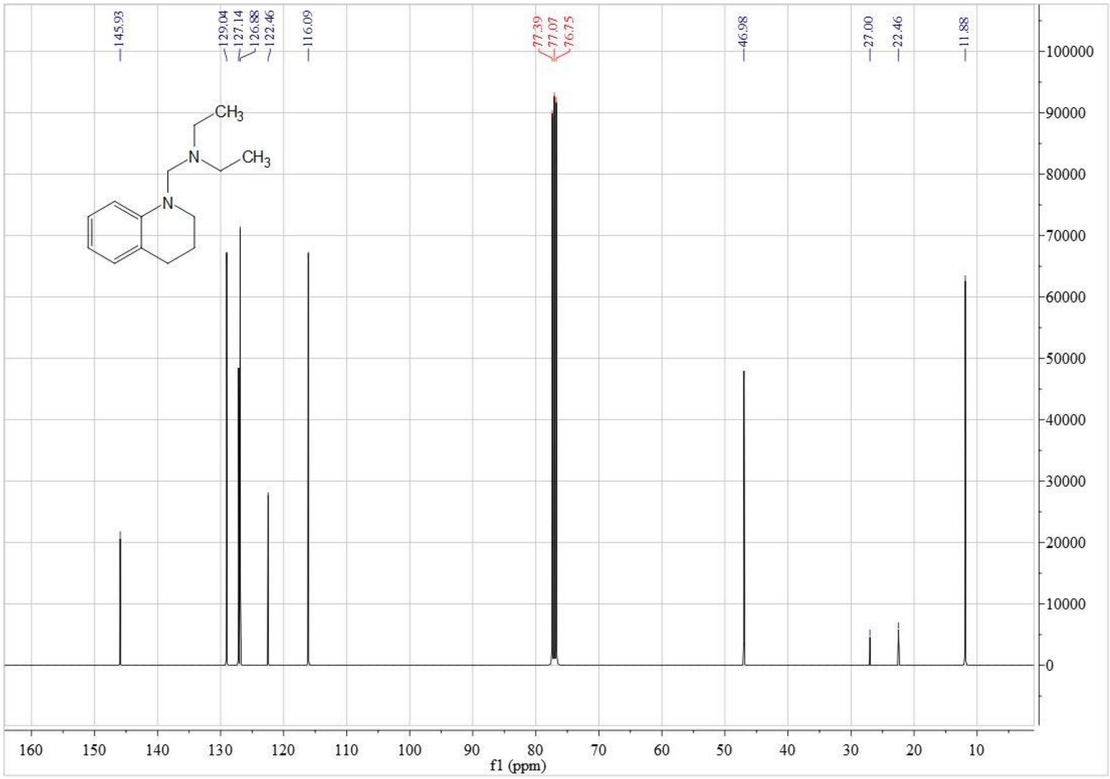

Figure S22: Carbon NMR of compound SF11

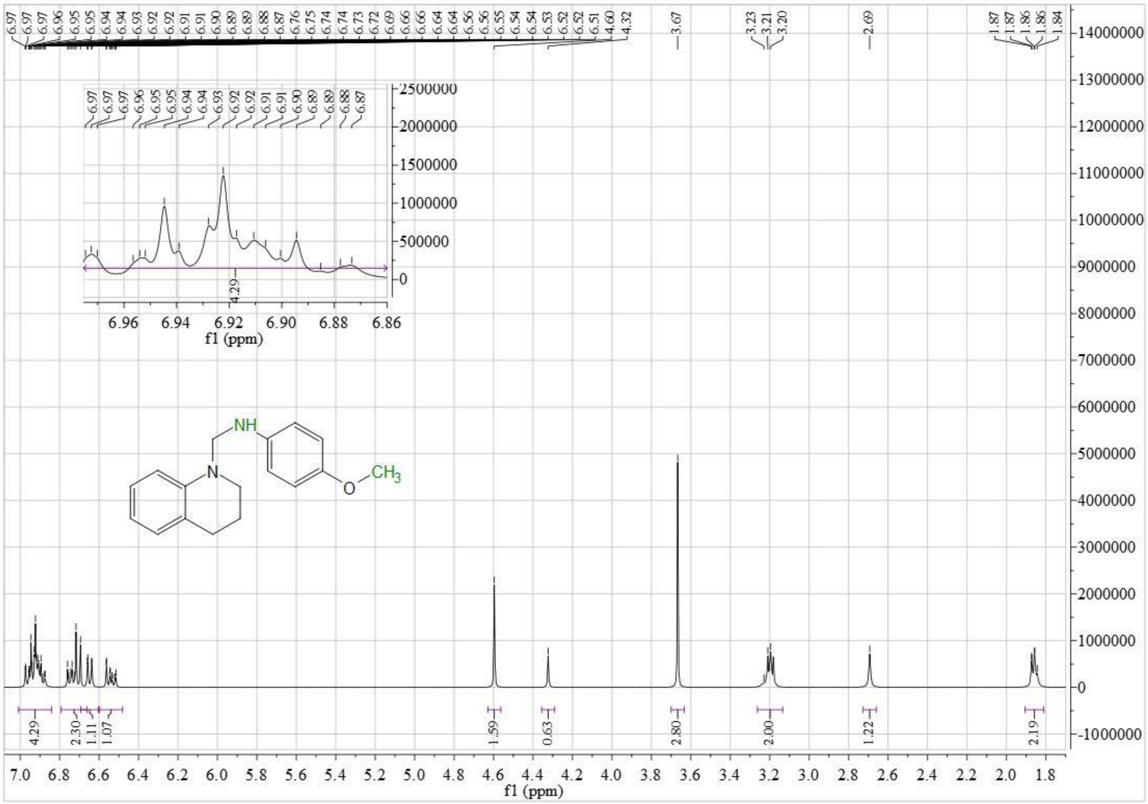

Figure S23: Proton NMR of compound SF12

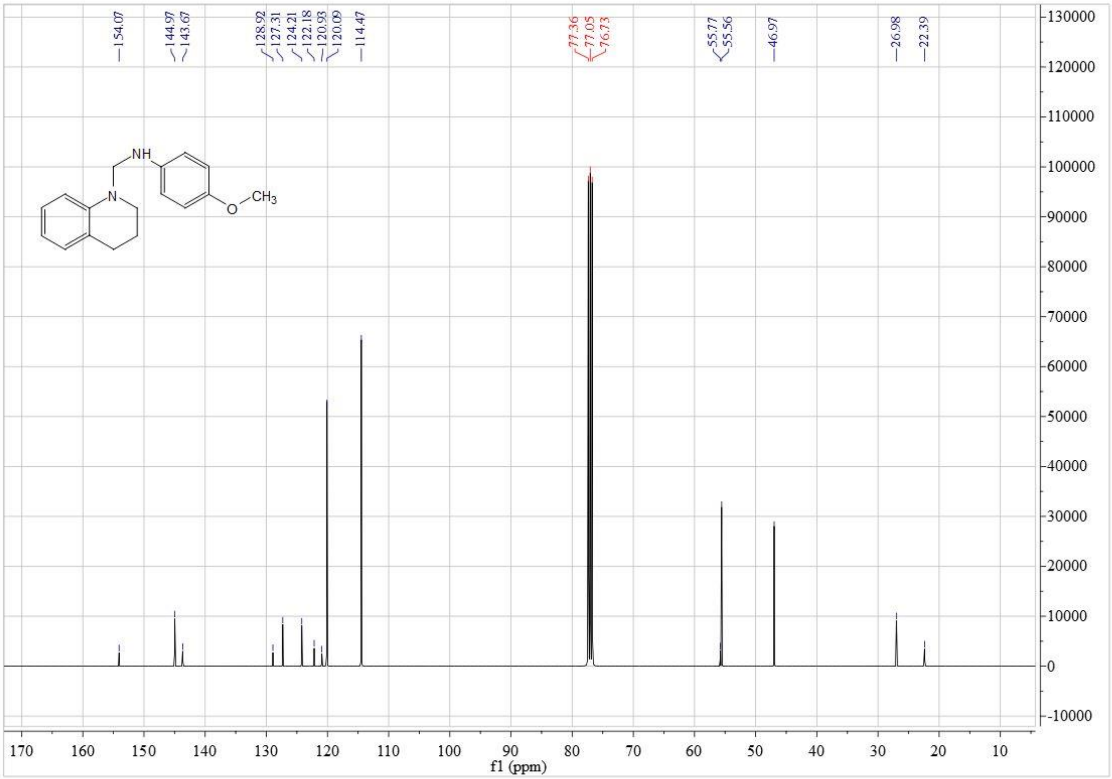

Figure S24: Carbon NMR of compound SF12

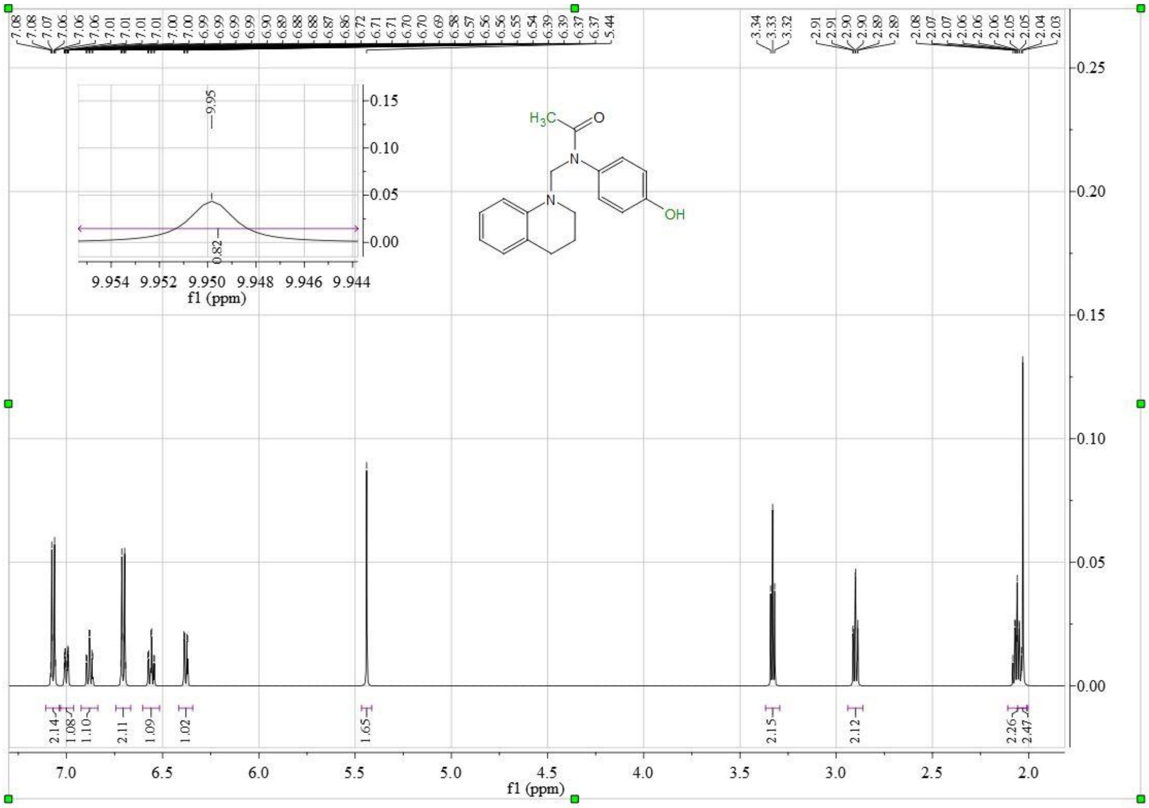

Figure S25: Proton NMR of compound SF13

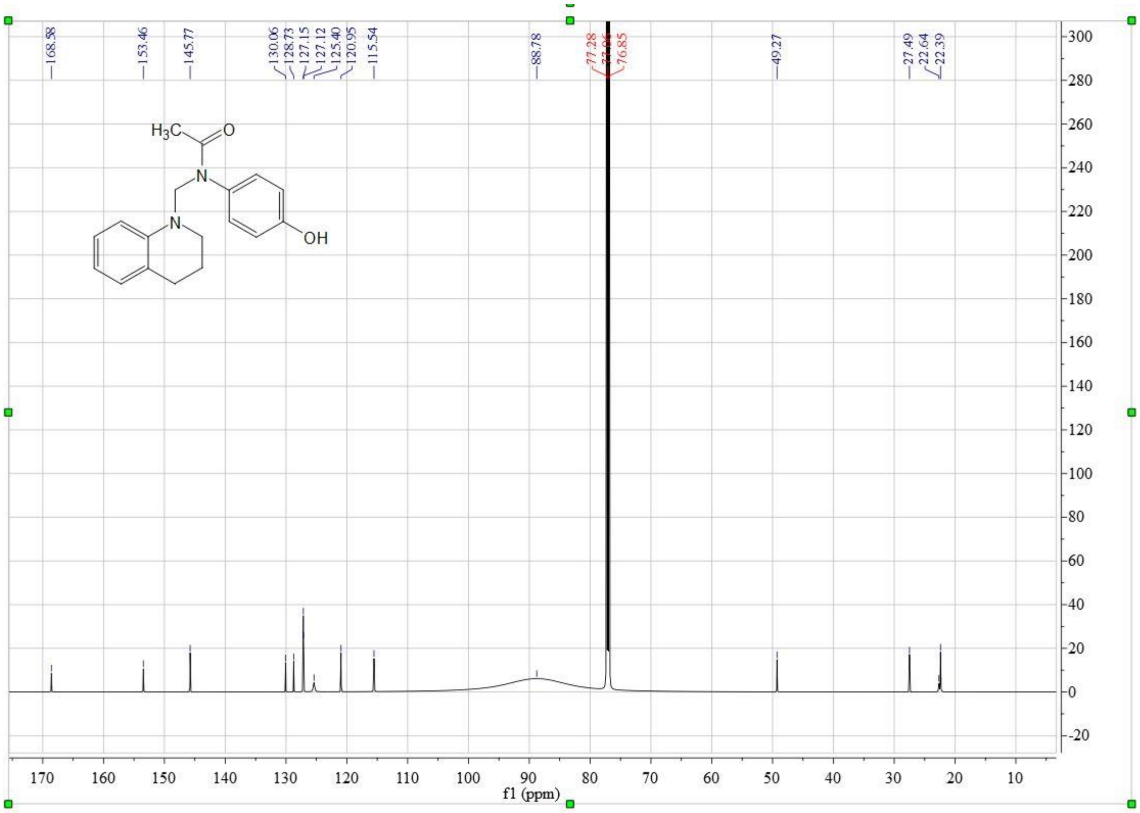

Figure S26: Carbon NMR of compound SF13
